# Chemokines in COPD: From Implication to Therapeutic Use

**DOI:** 10.3390/ijms20112785

**Published:** 2019-06-06

**Authors:** Pauline Henrot, Renaud Prevel, Patrick Berger, Isabelle Dupin

**Affiliations:** 1 Univ-Bordeaux, Centre de Recherche Cardio-thoracique de Bordeaux, U1045, CIC 1401, F-33604 Pessac, France; pauline.henrot@gmail.com (P.H.); renaud.prevel@hotmail.fr (R.P.); patrick.berger@u-bordeaux.fr (P.B.); 2 INSERM, Centre de Recherche Cardio-thoracique de Bordeaux, U1045, CIC 1401, F-33604 Pessac, France; 3 CHU de Bordeaux, Service d’exploration fonctionnelle respiratoire, CIC 1401, F-33604 Pessac, France

**Keywords:** COPD, chemokines, exacerbation, airway remodeling, chronic inflammation, gradient, biomarker

## Abstract

Chronic Obstructive Pulmonary Disease (COPD) represents the 3rd leading cause of death in the world. The underlying pathophysiological mechanisms have been the focus of extensive research in the past. The lung has a complex architecture, where structural cells interact continuously with immune cells that infiltrate into the pulmonary tissue. Both types of cells express chemokines and chemokine receptors, making them sensitive to modifications of concentration gradients. Cigarette smoke exposure and recurrent exacerbations, directly and indirectly, impact the expression of chemokines and chemokine receptors. Here, we provide an overview of the evidence regarding chemokines involvement in COPD, and we hypothesize that a dysregulation of this tightly regulated system is critical in COPD evolution, both at a stable state and during exacerbations. Targeting chemokines and chemokine receptors could be highly attractive as a mean to control both chronic inflammation and bronchial remodeling. We present a special focus on the CXCL8-CXCR1/2, CXCL9/10/11-CXCR3, CCL2-CCR2, and CXCL12-CXCR4 axes that seem particularly involved in the disease pathophysiology.

## 1. Introduction

Lung exposure to various types of noxious particles, such as those present in cigarette smoke, can lead to COPD. COPD is a common and devastating respiratory disease, characterized by a progressive airflow obstruction. COPD has become the third leading cause of global death by 2010, with 2.9 million deaths annually [1]. The Global Burden of Disease Study reports a prevalence of 251 million cases of COPD globally and an incidence of 95 million cases per year in 2016 [2]. In a prospective population-based cohort study with 25 years of follow-up (Rotterdam Study), the prevalence of COPD is 4.7% and the overall incidence is approximately 9/1000 per year [3]. The major risk factor is cigarette smoke, but there is a wide variety of interindividual responses for the same amount and duration of tobacco smoking. In total, the onset of the disease results from complex interactions between cumulative exposure to air pollutants and factors related to the individual himself, such as genetic factors [4], bronchial hyperresponsiveness [5], poor respiratory function some months after birth [6], or low lung function in early adulthood [7].

The chronic course of the disease is frequently worsened by recurrent episodes of acute worsening of respiratory symptoms, called acute exacerbations, most often related to viral or bacterial infections [8]. Acute exacerbations dramatically affect quality of life and worsen the natural history of the disease: Lung function decreases more rapidly in patients with frequent exacerbations, with an increased risk of death [9]. Current pharmacological treatments for COPD patients decrease exacerbation frequency by only up to 29% compared to a placebo either alone or in combination, but they do not have any significant effect on mortality, as shown by 5 large studies (TOwards a Revolution in COPD Health (TORCH) [10], Understanding Potential Long-term Impacts on Function with Tiotropium (UPLIFT) [11], Study to Understand Mortality and Morbidity in COPD (SUMMIT) [12], EFfect of Indacaterol Glycopyronium Vs Fluticasone Salmeterol on COPD Exacerbations (FLAME) [13], and InforMing the PAthway of COPD Treatment (IMPACT) [14]).

COPD is associated with an early activation of structural (epithelial) and innate immune cells (in particular, macrophages, neutrophils, and eosinophils) within the lung. These early events favor the accumulation of adaptive immune cells (CD4^+^ and CD8^+^ T cells and B cells) in the airways and the alveolar compartments, leading to chronic inflammation [15]. Other distinguishing feature of COPD includes bronchial remodeling and, in particular, peribronchial fibrosis. The origin of peribronchial fibrosis remains controversial: It has been recently suggested that circulating fibrocytes recruited into the lungs could play a role in this process, either directly or indirectly [16]. Although inflammation and remodeling are distinct processes, they are related; on the one hand, the infiltration of inflammatory cells into the lungs can influence cell survival, proliferation, migration and differentiation of structural cells, and on the other hand, the modification of lung architecture could also modify and perpetuate chronic inflammation. This has led some investigators to hypothesize that both chronic inflammation and bronchial remodeling should be targeted by treatment to slow down disease progression.

In this context, molecules of the chemokine family are of particular interest. Chemokines are small molecules (8 to 12 kDa), belonging to the family of cytokines. Because they are implicated in various biological functions such as chemotaxis [17], leukocyte degranulation [18], hematopoiesis [19] and angiogenesis [20], they represent therapeutic targets in numerous diseases, such as breast cancer [21], human immunodeficiency virus (HIV)/acquired immune deficiency syndrome (AIDS) [22], and atherosclerosis [23]. Multiple studies have now established a pivotal role for chemokines in COPD development and progression. This review attempts to highlight important recent advances in this field. In particular, we systematically reviewed chemokines modifications associated with COPD (Table 1), and based on the number of preclinical and clinical evidences as well as recent findings, we focus on the CXCL8-CXCR1/2, CXCL9/10/11-CXCR3, CCL2-CCR2, and CXCL12/CXCR4 axes that seem to be clearly associated with COPD pathophysiology. Research on chemokines and their receptors could pave the way for the development of new strategies of treatments which are urgently needed both to slow the natural course of the disease and to treat acute episodes of exacerbations.

## 2. General Considerations on Chemokines in COPD

### 2.1. Chemokines and their Receptors: Definition and Properties, in Relation with COPD

Chemokines are small molecules that belong to the large family of cytokines. Through their interaction with cell surface G-protein coupled receptors (GPCRs), they mediate various cellular processes, in particular, chemotaxis. Four groups of chemokines (CC, CXC, XC, and CX3C) are defined by the positions of sequentially conserved residues and by their quaternary structures [24]. All chemokines have a highly conserved tertiary structural fold, consisting of a flexible N-terminus and N-terminal loop, followed by a three-stranded β-sheet with a C-terminal α-helix. This conformation allows them to form oligomers, primarily homo, and heterodimers but also tetramers and higher order oligomers [25].

**Figure 1:**
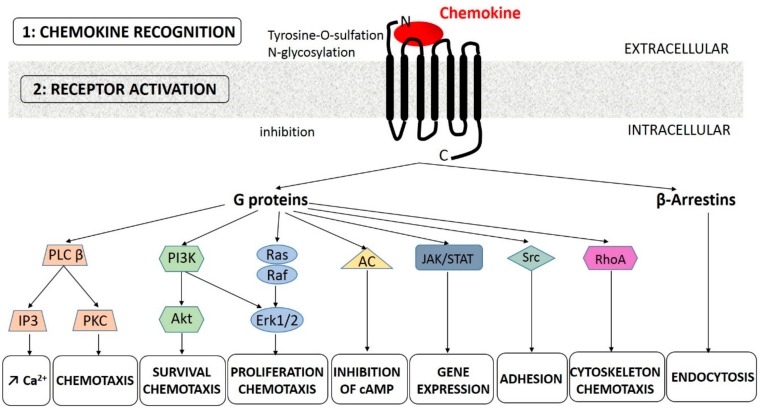
Chemokine-receptor interaction and the activation of downstream signaling pathways. Two main interactions between chemokines and their receptors are generally accepted: The N-terminal region of the chemokine binds in the pocket of the receptor transmembrane helical domain, while the N-terminal region of the receptor binds to a structural loop of the chemokine [37]. Evidence for more interactions has been reported [37]. Posttranslational modifications in the N-terminus part of chemokine receptor, such as tyrosine-O-sulfation and N-glycosylation, can affect this first binding step. A second activation-step then occurs that stabilize the receptor in an active conformation. Knowledge about this two-step mechanism and structural information of the chemokine—receptor interaction has been reviewed recently [38]. AC: Adenylate cyclase, C: C terminus part, IP3: Inositol trisphosphate, JAK/STAT: Janus kinase/signaling transducer and activator of transcription, Erk: Extracellular signal-regulated kinase, N: N terminus part, C: C terminus part, PI3K: Phosphoinositide 3-kinase, PKC, Protein kinase C, PLC: Phosphoinositide-specific phospholipase C.

So far, 20 chemokine GPCRs have been described [26]. Like chemokines, they are classified into 4 groups (CCR, CXCR, XCR, and CX3CR). Each chemokine can bind to several receptors of the same group and vice versa. Atypical receptors also exist [27]. At the structural level, the N-terminal part of some GPCR is involved in chemokine interaction, while the tripeptide ELR sequence (Glu-Leu-Arg) of some CXC chemokines was shown to be crucial for binding and activating GPCR. This has allowed the distinction between ELR^+^ CXC chemokines (CXCL1, 2, 3, 5, 6, 7, and 8), that promote angiogenesis, from ELR^−^ CXC chemokines (CXCL4, 9, 10, and 1) that have angiostatic properties [28]. Other residues such as the Arg-Phe-Phe-Arg-Glu-Ser-His sequence of CXCL12 within the N-loop between the 2 N-terminal cysteines are implicated in GPCR binding. Posttranslational modifications in the N-terminal part of GPCR, such as tyrosine-O-sulfation, participate in the specific interaction between the ligand and the receptor [29] (Figure 1). For example, CXCR4 sulfation at residues 7, 12, and 21 favors the binding of CXCL12 [30].

The binding of the chemokine on its receptor triggers the GDP/GTP exchange of coupled heterotrimeric Gi proteins and the dissociation of the β/γ subunits. These events induce the activation of Src family kinases, phosphoinositide-specific phospholipase C (PLC) β, and phosphoinositide 3-kinase (PI3K), with subsequent inositol trisphosphate (IP3)-mediated calcium mobilization and protein kinase C (PKC) activation (for reviews, see References [31,32]). Depending on the considered chemokine receptor, its surface expression, its density, and the cellular context, several downstream signaling pathways can be activated: the mitogen activated protein kinase (MAPK)/extracellular signal-regulated kinase (Erk) signaling pathway, the RhoA pathway, the Janus kinase (JAK)/signaling transducer and activator of transcription (STAT) pathway, and the PI3K/protein kinase B (Akt) pathway. Various biological effects can then be produced, including cell migration and chemotaxis, cell proliferation, and survival. In some cases, the binding of the chemokine does not trigger cell survival: Prolonged exposure to CXCL12 induces apoptosis in T cells [33], colorectal carcinoma cells [34], and acute myeloid leukemia cell lines [35]. Thus, in the context of CXCL12-rich bone marrow, CXCL12 may promote cell apoptosis rather than cell survival, especially if the other pro-survival signals are affected. Importantly, the activation of G proteins is rapidly attenuated by a negative feedback system, called desensitization, leading to β-arrestins mediated endocytosis of the receptor [31]. Chemokines receptors can also be regulated by other molecules. For instance, IL-6 regulates CCR5 expression. In a recent study, despite an increased CCR5 expression, monocytes of COPD patients exhibit decreased migratory ability [36].

Various chemokines are often produced at the target sites of leukocyte trafficking, and combinatorial effects could occur at different levels: Chemokines heterodimers/oligomers can modify biological activity, or each chemokine can act on its respective GPCR to trigger synergistic or antagonistic effect [39]. Chemokines can also interact with endogenous damage-associated molecular patterns (DAMPs), such as high mobility group box 1 protein (HMGB1), an alarmin that enhances the activity of CXCL12 [40] and that protects CXCL12 from degradation [41]. As cigarette smoke exposure and infections promote DAMPs released by damaged cells or by the immune system, this should be taken into account when exploring chemokine function in COPD.

Chemokines also bind strongly to glycosaminoglycans (GAGs), and this interaction can drive chemokines oligomerization [42], which is critical for subsequent biological effects. For example, monomeric and dimeric CXCL12 induce selective signal transduction pathways and differ in β-arrestin recruitment, and only the monomeric type promotes cell migration [30,43]. For some chemokines such as CCL2, CCL4, and CCL5, GAG binding and oligomerization are required to induce cell recruitment [44]. Specific changes of pulmonary extracellular matrix (ECM) are associated with COPD and occur in all lung compartments [45]. In COPD, the major changes described are a destruction of elastic fibers, as well as elevated levels of hyaluronan (belonging to the GAG family) and tenascin C with decreased level of decorin. Interestingly, an accelerated turnover of ECM proteins has been evidenced during acute exacerbations of COPD, which could further affect chemokine expression levels and localization patterns [46]. In addition, chemokines are the target of numerous proteases, such as MMP-1, 2, 3, 9, 13, and 14; cathepsin; elastase; and DPPIV/CD26 for CXCL12. Tissue decomposition in emphysema is usually described as a result of an imbalance between protease and antiprotease activities that could also have direct implication on chemokines degradation.

Of note, the conservation of the chemokine expression pattern between human and rodent models (essentially mice) has not been thoroughly investigated in COPD. However, some studies aiming to compare the expression of specific chemokines in other contexts have shown a poor correlation between human and mice in the basal state [47,48]. Therefore, conclusions drawn from murine models should be taken with caution.

### 2.2. Chemokines Gradients in COPD

Chemokine receptor expression allows the cell to sense and to respond to a concentration gradient. While chemokines and their receptors are well-described, literature on gradients is much sparser. One of the key problems is to measure *in vivo* the concentrations and their dynamics to characterize chemokine gradients. In addition, the microenvironment constantly evolves, leading to temporal and spatial modification of graded signals.

Another important distinction is to know whether the considered gradient is in the fluid phase or is a gradient of immobilized chemokines by their interaction with the ECM molecules, especially GAGs. Pioneer studies, such as those performed on CCL21 mediated dendritic cell migration [49], have provided *in vivo* pieces of evidence of directional migration along gradients of tissue immobilized chemokines. Because chemokines have short half lives in solution, soluble gradients may be more transient, but the cues may play a role at longer range that immobilized chemokines. In the context of COPD, both immobilized and soluble gradients probably coexist. While immobilized gradients are in principle more stable and robust that soluble gradients, both are probably differently modified by COPD exacerbation.

We also propose to distinguish intra-tissue gradients from inter-tissue gradients. At the organism level, inter-tissue gradients might play a critical role in orchestrating cell trafficking between bone marrow, lymph nodes, peripheral circulation, the lungs, and the heart. In peripheral circulation, chemokines are present both in solution and immobilized on the endothelial cell surfaces, where they can trigger leukocyte arrest and extravasation. In principle, these gradients should be evidenced by measuring chemokines concentration in different organs, as already done for CXCL12 in the context of idiopathic pulmonary fibrosis [50]. On the other hand, intra-tissular gradients, particularly intrapulmonary gradients, are probably mostly composed of chemokines bound to the ECM.

A consideration at the gradient level is key to understand *in vivo* effects. For example, neutrophil recruitment to the lungs is increased in response to mutant CXCL8 that binds less strongly to the GAGs [51]. This surprising effect has been explained by tissue-specific differences in GAG interactions: The mutant CXCL8 is less active in the peritoneum but more active in the lungs. Finally, the kinetics of gradient formation, strongly regulated by the kinetics of the interaction between chemokines and GAGs is another important point to consider [52]. This has important consequences during COPD exacerbation: Chemokines that bind to GAG with rapid kinetics will form gradients slowly and vice versa, leading to potential early and late effects.

## 3. Chemokines at the Stable State and During Exacerbation

COPD pathophysiology is related with small airways inflammation, which is associated with increased numbers of macrophages and T cells (mostly CD8^+^ T cells) and, in more severe disease, increased numbers of B cells within the parenchyma and neutrophils in the lumen [15]. In the respiratory tract, cigarette smoke and other irritants might activate alveolar macrophages and airway epithelial cells to release chemotactic factors that then attract circulating leukocytes to the lungs. Eosinophils, innate immune cells, can also be found in the blood and in lung tissue in a proportion of patients with COPD [53]. The natural history of COPD is often marked by periodic exacerbations, characterized with a worsening of the functional respiratory state, as well as an increased sputum production [54]. These exacerbations can be triggered by environmental (air pollution and meteorological effect) or infectious (bacteria and viruses) factors [8]. These factors can trigger the release of damage-associated molecular patterns (DAMPs) which, by binding to specific pattern recognition receptors (PRRs), induce the release of chemotactic factors by resident cells. Therefore, chemokines, produced by various cell types both in the lung and in the systemic circulation (see Table 1 for details), play a key role in orchestrating the chronic inflammation in the lungs of COPD patients.

Of note, the implication of several chemokines in COPD is still controversial. First, many chemokines seem to be associated with smoking status rather than the airflow obstruction characteristic of COPD [82]. For instance, a recent study found that circulating levels of the chemokines CXCL8, CCL4, and CCL22 attain the highest levels in healthy smokers compared to nonsmokers and COPD subjects [91]. The same finding has been observed with CCL17 which has increased mRNA levels in pulmonary cells and in the broncho-alveolar lung fluid (BALF) of COPD patients compared to nonsmokers controls but not compared to current or ex-smokers [92]. Overall, it is likely that some chemokines are increased in smokers compared to nonsmokers, but their levels seem to be further increased in smokers susceptible to develop COPD (for instance, see Reference [93] where CXCL8 levels are increased in smokers with emphysema).

Because some chemokines are continuously expressed while others are specifically induced during exacerbations, we chose here to distinguish chemokine function at a stable state and during an exacerbation. Table 1 presents the list of chemokines modifications associated with COPD, as well as secreting cells and resulting attracted cells. Chemokines receptors implicated in COPD, as well as their connection/interaction with cytokines have been reviewed recently [90]. We then develop hereafter the CXCL8-CXCR1/2, CXCL9/10/11-CXCR3, CCL2-CCR2, and CXCL12-CXCR4 axes that seem to be associated with COPD pathophysiology based on in vitro as well as *in vivo* findings. Of note, we choose here not to focus on the CCL11-CCR3 axis which might be another key player in COPD, in particular for eosinophil trafficking. Although eosinophil count is emerging as a biomarker, the role of eosinophils as mediator of disease remains to be fully elucidated [53].

### 3.1. The CXCL8-CXCR1/2 Axis

#### 3.1.1. At the Stable State

**Figure 2:**
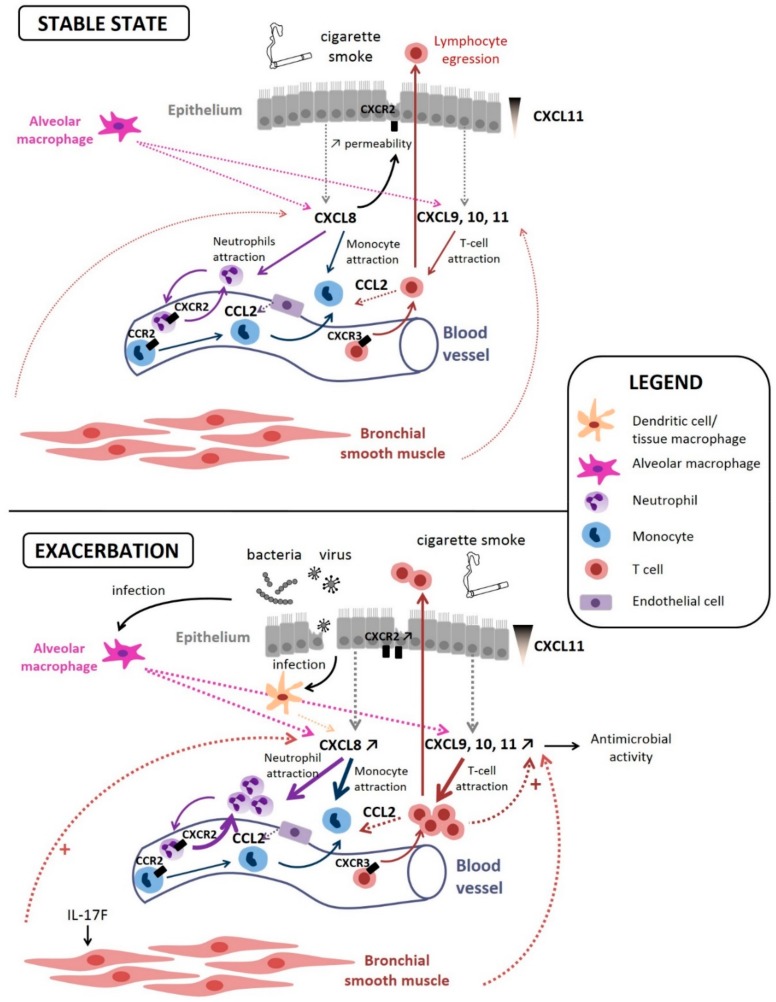
CXCL8-CXCR1/2, CXCL9/10/11-CXCR3, and CCL2-CCR2 axis implications at stable state and during an exacerbation in a COPD lung. At the stable state, CXCL8 (produced by alveolar macrophages, epithelial cells, and bronchial smooth muscle cells) binds on CXCR2 to attract circulating neutrophils and monocytes into the bronchial tissue as well as to increase epithelial cells permeability. CXCL9, 10, and 11 (produced by alveolar macrophages, epithelial cells, and bronchial smooth muscle cells) bind on CXCR3 to attract circulating T cells. A CXCL11 intra-epithelial gradient is responsible for lymphocyte egression towards the lumen. CCL2 (produced by T cells and endothelial cells) binds on CCR2 to attract monocytes. During an exacerbation, production of CXCL8 as well as CXCL9, 10 and 11 is enhanced, which increases the recruitment of monocytes and T cells into the bronchial tissue, participating to the immune (often triggered by microbial infection) response. Chemokines production by bronchial smooth muscle cells can be further enhanced upon IL-17F stimulation. The plain arrows (without other indications) show cell movements; the dotted arrows indicate chemokine secretion.

CXCL8 (IL-8) is one of the first intensively studied chemokines and a well-known mediator of neutrophilic inflammation [60,94,95]. It has first been identified as a Lipopolysaccharide (LPS)-stimulated monocyte-secreted factor that stimulated neutrophil exocytosis (granule release) and oxidative burst [96]. It is also the first identified chemokine to play a role in the recruitment of inflammatory cells in COPD: Increased concentrations of CXCL8 have been found in induced sputum of COPD patients compared with smokers with normal lung function and nonsmokers, and these increased CXCL8 levels have been linked with increased neutrophil numbers in sputum [60]. Since then, many studies have confirmed the role of CXCL8 in neutrophilic inflammation occurring in COPD airways. CXCL8 is thought to be produced by alveolar macrophages as well as bronchial epithelial cells [90] (Figure 2). in vitro studies have notably found a secretion by primary bronchial epithelial cell cultures exposed to diesel exhaust [97], as well as an increase in its production by the bronchial epithelial cell line (i.e., BEAS-2B) exposed to small particulate matter [98] and in another bronchial epithelial cell line (16-HBE) stimulated by HSP60 [99]. This production could stimulate the airway epithelium, leading to its contraction and an increased permeability to inflammatory cells [100]. Of note, CXCL8 is also thought to be produced by lung fibroblasts when stimulated by epithelial cell-derived IL-1α [61] but also by bronchial smooth muscle cells [62] (Figure 2). Some studies have been conducted on mice models and have found that the mouse equivalent of CXCL8 (keratinocyte-derived chemokine) is also increased in the lungs of mice exposed to cigarette smoke extracts (CSE) [101].

Overall, cellular crosstalk between alveolar macrophages and epithelial-secreted CXCL8 and CXCR2-expressing neutrophils contributes to COPD physiopathology.

#### 3.1.2. During Exacerbation

The role of CXCL8 in COPD exacerbations has been pointed out several times but not always with the same outcomes. The first study to measure CXCL8 levels during an exacerbation showed an increase in the sputum of exacerbating COPD patients compared to their basal state [102]. Of note, sputum CXCL8 levels correlate with the airways bacterial load and blood myeloperoxidase (a pro-inflammatory enzyme released by neutrophils) levels [103]. Another study showed an increase in serum CXCL8 levels from controls to stable, and exacerbation stage [104]. However, another large-scale study showed that serum CXCL8 is negatively associated with COPD moderate to severe exacerbations within the COPD Gene cohort [82]. Of note, this finding was only observed in one of the two cohorts investigated in this large-scale study. CXCL8 level was also associated with progressive emphysema. Overall, this discrepancy may be related with the fact that CXCL8 has been studied at different sites (serum *vs* sputum) and/or that the investigated cohorts were different. Finally, another team showed that consistent acute exacerbations (≥1 event per year for 3 years) were associated in logistic regression with higher CXCL8 concentrations in the SPIROMICS cohort [105].

The role of CXCL8 in COPD exacerbations is further supported by the correlation of sputum CXCL8 levels with the number of neutrophils [106]. Moreover, CXCR2 (but not CXCR1) mRNA is increased in bronchial epithelial biopsies in exacerbating compared to stable patients [58]. in vitro findings are consistent with these observations, since CXCL8 is upregulated in the human bronchial epithelial cell line (HBE-16) when stimulated by LPS [99].

Finally, CXCL8 is also associated with the IL-17 pathway. Indeed, IL-17F stimulation of human bronchial smooth muscle cells induces the transcription of the CXCL8 gene via the positive transcription elongation factor b (P-TEFb) composed of cyclin T1 and cyclin-dependent kinase 9 (CDK9). IL-17F is able to trigger CDK9 phosphorylation, thus enhancing the production of CXCL8 [107]. This sheds light on different mechanisms by which CXCL8 expression is activated during an exacerbation.

### 3.2. The CXCL9/10/11-CXCR3 Axis

#### 3.2.1. At the Stable State

CXCL9, CXCL10, and CXCL11 are mainly produced by alveolar macrophages [108] but also by bronchial epithelial cells [109,110] and bronchial smooth muscle cells [71] (Figure 2). The affinity for their common receptor (CXCR3) is increasing from CXCL9 to CXCL11, suggesting a hierarchy of affinity [111]. Their concentrations are increased in the induced sputum of COPD patients and correlates with the number of neutrophils [68]. However, CXCR3 agonists are mainly known for their ability to attract both Th1 and CD8^+^ T lymphocytes (or type 1 cytotoxic T cells) [112]. Peripheral blood mononuclear cells (PBMC) of COPD patients have an enhanced migratory response towards CXCL9, CXCL10, and CXCL11, compared with PBMC from nonsmokers [113], which was apparently not due to an increased number of receptors at the cell surface. Isolated lymphocytes or monocytes from COPD patients do not show enhanced migration towards these chemokines, suggesting that lymphocytes in PBMC are stimulated by the presence of other leukocytes in the PBMC fraction. This concurs with another report that only activated lymphocytes migrate towards CXCL11 [114].

Furthermore, CXCR3 expression is induced on activated Th1 and CD8^+^ lymphocytes and is thought to be involved in the recruitment of these cells to the sites of inflammation in COPD patients [72]. Moreover, a decline of lung function in COPD patients is associated with a high percentage of T cells that express CXCR3 [115], suggesting that CXCL9, CXCL10, and CXCL11 may be involved in the recruitment of T cells and the subsequent immune mediated lung damage observed in COPD, through the production of perforins and granzyme B [116].

By contrast, a CXCL11 gradient in the lungs of COPD patients is also associated with the resolution of pulmonary inflammation. The recruitment of effector T cells to mucosal surfaces, such as bronchial epithelium, is important to provide defense against airway pathogens, but the migration of the lymphocytes from the epithelium to the airways lumen, called “egression”, is also physiologically crucial to avoid detrimental accumulation of leukocytes within the interstitium. The polarized secretion of CXCL11 at the apical side of bronchial epithelial cells produces a basal-to-apical gradient that triggers lymphocyte egression (Figure 2). This gradient is upregulated in COPD patients [117], resulting in more egression. Thus, the upregulation of CXCL11 production could also be seen as a defense mechanism. This migration may also depend on the underlying stimulus and may vary as the inflammatory responses evolves.

Overall, CXCL9/10/11 produced by macrophages and bronchial epithelial cells act on CXCR3^+^ cells to recruit inflammatory cells into the lungs, such as T lymphocytes, but CXCL11 seems to be also involved in lymphocyte clearance, emphasizing the possible dual role of chemokines in COPD.

#### 3.2.2. During Exacerbation

CXCL10 has been found elevated in the sputum of COPD patients during an exacerbation compared to values after recovery [118]. CXCL10 is also elevated in the serum from COPD patients suffering from a rhinovirus-induced exacerbation compared to control serum [73] and serum CXCL10 has been proposed as a biomarker of virus-associated exacerbations of COPD [119]. in vitro, CXCL9, CXCL10, and CXCL11 are overexpressed when bronchial epithelial cells are stimulated with IFN-γ [109,110]. Moreover, a release of these three chemokines can be potentiated by the synergistic interactions between TNF-α and IFN-γ [110,120]. Elevated levels of TNF-α have been found *in vivo* in the airways of COPD patients [60], and thus, the expression of these three chemokines by structural cells of the airways is likely to drive lymphocyte recruitment during an exacerbation. Finally, T lymphocytes and IFN-γ-stimulated mononuclear cells also produce these 3 chemokines, and it has been shown that these molecules can also have anti-microbial properties [121].

### 3.3. The CCL2-CCR2 Axis

#### 3.3.1. At the Stable State

CCL2 activates CCR2 mainly expressed on monocytes and T cells. In the lungs, CCL2 is mainly produced by alveolar macrophages, T cells, and endothelial cells, and this increase results in local monocyte recruitment (Figure 2). CCL2 levels have been found to be increased in COPD patients compared to healthy controls in various studies and organs, such as whole blood, induced sputum, and *in situ* lung tissue (see Table 1). Of note, polymorphisms in the CCL2 gene, as well as in that of CCR2 have been found associated with COPD development [122].

#### 3.3.2. During an Exacerbation

CCL2 has been implicated in a systemic inflammatory response (with elevated CCL2 serum level) which may be due to a virus-induced activation of macrophages in virus-infected COPD patients compared to healthy controls [123]. Furthermore, a mouse model of COPD exacerbation (porcine pancreatic elastase (PPE) and LPS-triggered) used to determine the role of IFN regulatory factor III (IRF3) showed a decrease in CCL2 mRNA transcripts in the lungs of IRF3-/- mice. This finding suggests that CCL2 is implicated in LPS/bacteria-induced COPD exacerbation [124].

### 3.4. The CXCL12-CXCR4 Axis

#### 3.4.1. At the Stable State

The CXCL12-CXCR4 axis also represents an important chemotactic axis for numerous cells, such as T cells, monocytes, immature B cells, bone marrow-mesenchymal stem cells (BM-MSCs), endothelial progenitor cells (EPCs), and fibrocytes. CXCL12 is expressed by various cells, such as inflammatory cells, bronchial epithelial cells, hematopoietic cells, endothelial cells, and perivascular stromal cells [125,126] and in different tissues, such as the bone marrow, the lungs, and the blood vessels. CXCL12 directs stem-cell homing [127] and mediates angiogenesis [128]. Importantly, CXCL12 also binds to CXCR7, a receptor which has been more recently described [129].

Until recently, the role of the CXCL12-CXCR4 axis was unexplored in COPD. In mice exposed to cigarette smoke during 6 months, the level of CXCL12 is decreased in the lungs [74]. In COPD patients, both the number of CXCR4 positive circulating fibrocytes and the blood level of CXCL12 are unchanged at the stable state [76]. However, we very recently demonstrated that bronchial fibrocytes are increased in both distal and proximal airways of COPD patients at a stable state compared to controls [130]. The level of proximal bronchial fibrocytes is correlated with both a loss in lung function (i.e., FEV1 decrease) and bronchial thickness assessed by CT scan [130]. In addition, the level of distal bronchial fibrocytes is correlated with both functional obstruction (i.e., FEV1/FVC decrease) and air trapping assessed by mean lung attenuation during expiration assessed by CT scan [130]. It is thus tempting to speculate that these tissue fibrocytes could participate in tissue repair and bronchial remodeling. By contrast, the levels of both CXCR4 and CXCL12 mRNA are decreased in BM-MSCs of COPD patients [75], suggesting that their chemotactic properties are altered. The migratory and repair capacities of EPCs from COPD patients are also impaired, which could be related to a decreased expression of CXCR4 [131]. An alteration of the CXCR4/CXCL12 axis at the stable state seems, therefore, associated with stem cell senescence and defect in repair function in COPD.

#### 3.4.2. During Exacerbation

The level of CXCR4 expressing fibrocytes is enhanced in the blood of COPD patients during an exacerbation, and the chemotactic properties of these cells in response to CXCL12 are also increased [76]. This is apparently not due to a modification of CXCR4 expression level, as the total amount of CXCR4 is not modified in these cells [76]. This is also not related to the blood level of CXCL12, which is not increased during an exacerbation [76]. Such an increased fibrocyte migratory capacity during exacerbation may thus participate to their bronchial infiltration in the airways of COPD patients, even at a stable state (i.e., after exacerbations) [130]. In an influenza-infected mouse model, it has been shown that the neutrophils early recruited within the tissues leave CXCL12-containing trails behind [132]. CXCL12 derived from neutrophils but not from epithelial cells is critical for virus-specific CD8^+^ T cell recruitment [132]. Altogether, this suggests that the CXCL12-CXCR4 axis is modified during an exacerbation, providing one of the inflammatory cues necessary to guide immune cells into inflamed tissues.

## 4. Chemokines in COPD: Therapeutic Implications

### 4.1. Chemokine Levels as Biomarkers in COPD

Biomarkers in COPD are essential to predict the risk of developing the disease among people exposed to risk factors or to predict the severity of the COPD course. Lungs being at the first line of exposure to airborne particles, lungs biomarkers seem the most fitted option. Sampling can be done in the exhaled breath condensate (easily accessible but low protein content and low reproducibility), induced sputum (noninvasive but requires expertise and time), BALF, lung brushes, and biopsies (the most accurate but all invasive and expensive) [82].

Another option is to measure chemokines level in the blood, hypothesizing that COPD is a systemic disease or at least has features of a systemic disease. Of note, several protein biomarkers measured in the blood are already known: For instance, fibrinogen and C-reactive protein are correlated with COPD severity and risk of exacerbations [133,134]; soluble receptor for advanced glycation end-products (sRAGE) is inversely correlated with emphysema and airflow obstruction [135]; surfactant protein D (SP-D) has been associated with COPD and emphysema [136,137]; and club cell-16 (CC16) might be correlated with airflow obstruction and emphysema [137,138]. The ECLIPSE study has been a breakthrough concerning the assessment of biomarkers in COPD (see Reference [139] for an exhaustive review). Among chemokines, serum CCL18 has been identified as associated with an increased risk of cardiovascular hospitalization or mortality [86]. We present hereafter recent data concerning chemokines we have focused on in the previous part.

#### 4.1.1. CXCL8 as a Biomarker of Disease Severity

According to Baker et al., CXCL8 belongs to a category of molecules classically associated with cellular senescence, mirroring many inflammatory diseases such as COPD [140]. Indeed, CXCL8 is independently associated with severe COPD, notably worse airflow obstruction (FEV1% and FEV1/FVC), and with a progression of CT assessed emphysema over 5 years [82].

CXCL8 is also used as a biomarker in murine models of COPD (for instance, to validate the efficiency of a ghrelin-based therapy in a murine model of emphysema [141]).

#### 4.1.2. CXCL10 as a Genetic Biomarker of Disease Susceptibility

Polymorphisms in CXCL10 gene promoter could also contribute to susceptibility to COPD: The “rs56061981” single nucleotide polymorphism (SNP) is significantly associated with a reduced risk of developing COPD, and the “rs56216945” SNP is associated with an increased risk; the implicated mechanism could be an alteration of monocyte recruitment [142].

Future research should aim to assess the predictive value of other important chemokines (CCL2, CXCL12…) to determine biomarker combinations in order to better predict personalized risk, as well as to develop new techniques to better assess biomarkers in non-invasive lung samples.

### 4.2. Chemokines or Chemokine Receptors Neutralization in COPD

As several chemokines are implicated in COPD, the neutralization of chemokines and/or their receptors has received considerable interest. Most strategies directly antagonize the GPCR (for excellent review, see Reference [108]). Although very successful in some cases, attested by the example of plerixafor [143] and maraviroc [144], the respective antagonists of CXCR4 and CCR5, most of the pharmacological agents that target chemokine receptors inhibit both G protein signaling and β-arrestins-mediated endocytosis, promoting receptor accumulation at cell surface, a process called “antagonist tolerance” [145]. As a result, the efficacy of this type of antagonist is reduced after a long time of treatment. Other molecules, called “biased antagonists”, selectively inhibit G protein signalization without preventing β-arrestins recruitment to the receptor. These biased antagonists could offer in the future a solution for patients developing tolerance. Although in principle more complicated because chemokines are often considered too small and to have too shallow surfaces to be “druggable”, recent successes in drug discovery show that chemokines can also be targeted and neutralized [146]. Other approaches include interfering with chemokine-GAG interaction [42] and designing specific chemokine heterodimer agonists [147].

#### 4.2.1. The CXCL8-CXCR1/2 Axis as Therapeutic Target

Because neutrophils are often described as the main effector cells of tissue destruction, blocking CXCL8 was expected to lower neutrophilic inflammation and to slow down disease progression. Antibody-mediated blockade of CXCL8 partially inhibits sputum-induced neutrophil chemotaxis [57]. Targeting CXCL8 with an antibody only slightly improves dyspnea in COPD patients [148]. This modest effect can be probably explained by the fact that the antibody only blocks free and not bound CXCL8 and by the existence of other neutrophil chemotactic factors, such as leukotriene B4. Interfering with CXCL8 binding property with GAG could represent an alternative strategy: TNF-stimulated gene/protein-6 (TSG-6) inhibits neutrophil chemotaxis through a direct interaction between TSG-6 and the GAG binding site of CXCL8, which prevents the association of CXCL8 with heparin [149].

Neutralization of the CXCL8 receptors might be more promising. A specific CXCR2 antagonist, SB-332235, decreases neutrophil infiltration into the lungs in a rat model of acute cigarette smoke exposure [150]. In a similar mouse model, SCH-N, an orally active small molecule inhibitor of CXCR2 also lowers neutrophilic inflammation in the BALF, lung neutrophil infiltration and the level of tissue damaging enzymes [151]. In a phase II study, a CXCR2 antagonist, MK-7123, significantly improves the FEV1 and time to the first exacerbation, and it reduces inflammation after 6 months of treatment [152]. Another CXCR2 antagonist, danirixin, has been shown to improve respiratory symptoms and health status of treated patients [153] https://www.zotero.org/google-docs/?s0lRNK. Of note, although preclinical evidences are lacking due to species differences in CXCR1, dual CXCR1/2 therapy may be even more effective than selective CXCR2 antagonists [154]. A CXCR1/2 antagonist given orally for 3 days appears to markedly reduce neutrophils and macrophages counts in sputum after inhaled LPS challenge in healthy subjects [155], making it likely that these drugs may reduce pulmonary inflammation in patients with COPD.

#### 4.2.2. The CXCL9/10/11-CXCR3 Axis as a Therapeutic Target

Targeting CXCL10 seems an interesting option. An in vitro study has shown that CXCL10-neutralizing antibody can inhibit CSE-induced cell necrosis and activation of inflammatory cytokines in the 16HBE cell line [79]. In the same study, mice treated with CXCL10 neutralizing antibody did not develop COPD features (weight loss; reduction of lung function, increased levels of IL-6, and CCL2 in BALF and lung homogenate) as compared to cigarette smoke-exposed mice. Antagonists of the receptor CXCR3 have been developed [156], but so far, none of them has been tested yet in clinical trials for COPD.

#### 4.2.3. The CXCL12-CXCR4 Axis as a Therapeutic Target

The CXCL12-CXCR4 axis is another attractive target, and various strategies of neutralization have already been developed, such as locking CXCL12 in an homodimer structure [157], neutralizing CXCL12 [158,159,160], interfering with GAG binding properties of CXCL12 [161], and antagonizing CXCR4 by small molecules (for review, see Reference [162]) or blocking antibodies [21]. So far, plerixafor (also called AMD3100; brand name Mozobil) is the only FDA-approved CXCR4 antagonist. It is used to mobilize stem cells from the bone marrow for autologous stem-cell transplantation in patients with multiple myeloma or non-Hodgkin’s lymphoma [143]. In a recent study regarding WHIM syndrome, patients have been treated daily by plerixafor at low dose for at least 19 weeks [163], opening perspectives for chronic treatment.

**Figure 3:**
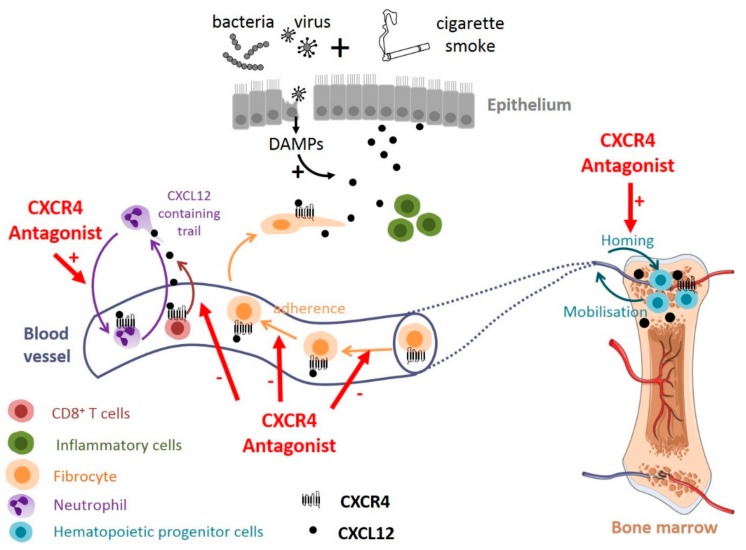
Potential effect of targeting CXCL12/CXCR4 axis in COPD. CXCR4 antagonists might act at various levels in COPD: they could promote neutrophil demargination from the lungs and inhibit T-cells and fibrocytes recruitment into bronchial tissue. CXCR4 antagonists may also contribute to maintaining the pool of hematopoeitic progenitor cells in the bone marrow, available for tissue repair. DAMPs: damage-associated molecular patterns.

Recent evidences suggest that targeting the CXCL12-CXCR4 axis may be promising for COPD treatment. In a mouse model of cigarette smoke exposure, the intermittent administration of plerixafor decreases emphysema damages, without affecting CXCL12 level and inflammation in BALF [74]. Plerixafor has a protective effect on the number of hematopoietic progenitor cells (HPCs) in the bone marrow following chronic smoke exposure [74] https://www.zotero.org/google-docs/?Ltp2HU, suggesting that increased HPCs available for lung repair might explain, at least partially, the beneficial effect of plerixafor (Figure 3). How plerixafor increases the number of HPCs remains unknown, but it could be explained by the good efficiency of marrow homing of plerixafor mobilized HPCs [164], or it could interfere with CXCL12-mediated apoptosis that have been described in various cells [33,34,35].

Plerixafor also significantly reduced the in vitro plasma-induced migration of fibrocytes purified from the blood of exacerbating COPD patients [76], suggesting that the CXCL12-CXCR4 axis could participate to the recruitment of fibrocytes to the lungs during COPD exacerbations [130]. Antagonizing CXCR4 may therefore reduce the tissular content of fibrocyte-derived fibroblasts and myofibroblasts and subsequent fibrosis, as shown in other clinical settings such as idiopathic pulmonary fibrosis [50] (Figure 3). We can speculate that this type of treatment could interfere not only with fibrocyte migration but also with their adherence to vessels, as CXCR4 activation has been shown to enhance β1 integrin function in melanoma cells, resulting in increased adhesion to endothelial cells [165].

In mice, an acute treatment with a high dose of plerixafor has also been shown to promote neutrophil demargination from the lungs [166]. In an influenza-infected mouse model, plerixafor treatment as well as genetic deletion of CXCL12 in neutrophils recapitulates the effect of neutrophil depletion on CD8^+^ T cell recruitment [132]. Thus, CXCR4 antagonists may also reduce lung inflammation in COPD in which neutrophils and cytotoxic CD8^+^ T lymphocytes are implicated (Figure 3). Although one could expect that this type of treatment could result in reduced CD8^+^ T cell effector function and could delay the clearance of the virus, it seems that T cell priming and expansion is not altered in this mouse model [132].

In addition to its effect on the lung and the bone marrow, plerixafor could have a protective effect on the heart: a single injection of plerixafor after myocardial infarction preserves mouse cardiac function by mobilizing the endothelial progenitor cells from bone marrow into peripheral blood [167]. This effect seems to be dependent on MMP-9 up-regulation, mediated by plerixafor-induced VEGF. Similar findings were found in a rat model of myocardial infarction [168]. As cardiovascular diseases are the most frequent comorbidities in COPD patients, it reinforces plerixafor interest as therapeutic option for COPD. Nevertheless, it should be emphasized that most of the data obtained in mice regarding plerixafor effect were obtained using acute treatment with high dose of plerixafor (usually, 5 mg/kg), and it is therefore possible that chronic exposure with a lower dose in humans may not produce the same effects.

In total, although potential detrimental effects can be expected, antagonizing the CXCL12-CXCR4 axis seems to represent an important opportunity to treat COPD, as recapitulated in the Figure 3.

## 5. Conclusions

The study of chemokines has led to substantial progress in our understanding of COPD pathogenesis and has major implications for potential COPD treatment. The role of chemokines in orchestrating the traffic of immune cells in COPD is now well-recognized, but additional key functions are emerging, albeit their direct contribution to COPD remains to be fully explored. Building novel tools and models to dissect these functions and to assess their roles in COPD remains a major goal for the field. In particular, deciphering the role of chemokines at the gradient level in bone marrow, lymph nodes, peripheral circulation, lungs, and heart will be important to grasp their logic and the potential beneficial and detrimental effects of chemokines or chemokine receptors neutralization. Finally, as COPD is a heterogeneous disease, we strongly believe that selecting subtypes of patients for future clinical trials may improve success in therapeutic development.

## Figures and Tables

**Table 1:** Chemokines implicated in Chronic Obstructive Pulmonary Disease (COPD).

Attracting Cells	Expressing Cells	Chemokine	Receptor	Studied Site/Organ	Model	Result	Ref.
Neutrophils > monocytes	Macrophages, mast cells	CXCL1 (GROalpha)	CXCR2 > CXCR1	Sputum	Human	Increased in COPD compared to IP	[55,56]
Neutrophils > monocytes	Macrophages, mast cells	CXCL2 (MIP-2, GRObeta)	CXCR2	BALF	Mouse	Increased in COPD compared to control	[57]
Neutrophils > monocytes	Alveolar macrophages, epithelial cells, platelets	CXCL5 (epithelial neutrophil-activating peptide 78)	CXCR2	Bronchial epithelium	Human	Increased at the mRNA level in severe exacerbatory COPD patients compared to stable COPD patients and controls	[58]
Neutrophils > monocytes	Inflammatory cells, fibroblasts, endothelial cells, platelets	CXCL7 (truncation product of CTAP-III)	CXCR2	Bronchial mucosa	Human	Increased number of CXCL7+ cells/mRNA level in stable severe COPD patients compared to healthy controls	[59]
Neutrophils > monocytes	Neutrophils, epithelial cells, macrophages, fibroblasts, airway smooth muscle cells	CXCL8 (IL-8)	CXCR1 CXCR2 CCR2	Lung fibroblasts	Human	Increased	[58,60,61,62,63,64,65,66,67]
				Blood		Increased in COPD compared to asthma	
				Induced sputum		Increased in COPD patients compared to controls (smokers and nonsmokers) and asthma	
				Lung		Increased in bronchiolar epithelium at the mRNA level	
Th1 lymphocytes, Tc1 lymphocytes, B lymphocytes	Macrophages, dendritic cells, bronchial epithelial cells	CXCL9 (MIG)	CXCR3	Lung section	Human	CXCL9 expressed in and around lung lymphoid follicles; - CXCR3 expressed in lung lymphoid follicles, correlated with GOLD stage, inversely correlated with FEV1	[68,69,70]
				Sputum	Human	Increased in the sputum of patients with COPD when compared with nonsmokers (but not smokers without obstruction)	
				Bone marrow	Guinea pig	Decreased at the mRNA level in the bone marrow of CS-exposed guinea pigs compared to controls	
Th1 lymphocytes, Tc1 lymphocytes, B lymphocytes	Macrophages, dendritic cells, bronchial epithelial cells	CXCL10 (IP-10)	CXCR3	Lung section	Human	CXCL10 expressed in and around lung lymphoid follicles; also in bronchiolar epithelium, airway smooth muscle cells	[68,69,71,72,73]
				Serum		Elevated in COPD patients compared to controls	
				Sputum		Increased in the sputum of patients with COPD when compared with nonsmokers (but not smokers without obstruction)	
Th1 lymphocytes, Tc1 lymphocytes, B lymphocytes	Macrophages, dendritic cells, bronchial epithelial cells	CXCL11 (I-TAC)	CXCR3	Sputum	Human	Increased in the sputum of patients with COPD when compared with nonsmokers (but not smokers without obstruction)	[68]
BM-MSCs, EPCs, HPCs, lymphocytes, fibrocytes	Inflammatory cells, epithelial and endothelial cells, perivascular stromal cells, BM-MSCs,	CXCL12 (SDF-1)	CXCR4, CXCR7	Bone marrow	Human	Reduced in COPD BM-MSCs compared to control subjects (mRNA level)	[74,75,76]
				Blood	Mouse	Enhanced chemosensitivity to CXCL12 of fibrocytes from exacerbating COPD level of CXCL12 decreased in CS-exposed murine lungs	
Naïve B cells	Follicular dendritic cells, B cells	CXCL13	CXCR5	Serum	Human	Decreased in the serum of BMS-COPD subjects compared to controls	[77]
Leucocytes, mononuclear cells	Endothelial, epithelial, and smooth muscle cells; macrophages, dendritic cells, B and T cells, and platelets (transmembrane chemokine)	CXCL16	CXCR6	Blood	Human	Percentage of CXCL16 (and CXCR6) expressing platelets is increased in COPD patients compared to controls	[78]
Monocytes > T lymphocytes, fibrocytes	Alveolar macrophages, T cells, endothelial and epithelial cells	CCL2 (MCP-1)	CCR2	Blood	Human	Increased in COPD with prevalent emphysema compared to control subjects	[56,65,66,79,80]
				Sputum	Human	Increased in COPD compared to nonsmokers and healthy smokers	
				Lung	Human	Increased in bronchiolar epithelium at the mRNA level	
				BALF and lung homogenate	Mouse	Increased in CSE-treated mice compared to control group	
Macrophages; Th2 lymphocytes	Macrophages, lymphocytes, fibroblasts, epithelial cells	CCL3 (MIP-1alpha)	CCR1, 4, 5	Induced sputum	Human	Increased in COPD patients compared to controls	[81]
Macrophages, neutrophils and dendritic cells; memory T cells; basophils, eosinophils; fibrocytes	Endothelial cells, smooth muscle cells; T lymphocytes, epithelial cells	CCL5 (RANTES)	CCR1, 3, 5	Bronchial mucosa	Human	Increased number of CCL5+ cells/mRNA level in stable severe COPD patients compared to healthy controls	[59,68]
				Sputum		Increased in the sputum of patients with COPD when compared with nonsmokers (but not smokers without obstruction)	
Eosinophils; Th2 lymphocytes	Epithelial cells, endothelial cells, T lymphocytes, macrophages, eosinophils	CCL11 (Eotaxin-1)	CCR3	BALF	Human	Increased in BALF of COPD with a bronchodilator response, and correlated with emphysema	[82,83,84,85]
				Blood		Increased in COPD (particularly rapidly progressive) compared to control subjects, and associated with decreased FEV1% and FEV1/FVC ratio	
				Lamina propria		Number of eotaxin+ and CCR3+ cells significantly higher in exacerbated COPD compared to healthy subjects	
T cells, monocytes;Eosinophils; endothelial cells	Basophils, lung leucocytes, alveolar macrophages, airway smooth muscle cells	CCL15	CCR1 > CCR3	Serum	Human	Decreased in the serum of BMS-COPD subjects compared to controls	[77]
TH2 lymphocytes	Dendritic cells, activated Langerhans cells, airway epithelial cells	CCL17 (TARC)	CCR4	Serum	Human	Decreased in the serum of BMS-COPD subjects compared to controls rs9302690 SNP significantly associated with higher CCL17 levels in COPD patients	[77,82]
T lymphocytes, immature dendritic cells	Dendritic cells, monocytes, alveolar macrophages	CCL18 (PARC/MIP-4/AMAC-1/DC-CK1/SCYA-18)	CCR8	Serum	Human	Increased in COPD compared to non-obstructive smokers and never smokers	[86,87]
Naïve T lymphocytes, mature dendritic cells	Fibroblasts	CCL19	CCR7	Bone marrow	Human	Reduced in COPD BM-MSCs compared to control subjects (mRNA level)	[75]
Dendritic cells; neutrophils, lymphocytes	Airway epithelial cells, macrophages	CCL20 (MIP-3alpha)	CCR6	Total lung and induced sputum	Human	Increased at the mRNA level in total lung and at the protein level in induced sputum, compared to never smokers and smokers without COPD	[88]
T lymphocytes, mature dendritic cells	Lymphatic endothelial cells	CCL21	CCR7	Bone marrow	Human	Reduced in COPD BM-MSCs compared to control subjects (mRNA level)	[75]
Memory T lymphocytes	Epithelial cells? (highly produced by keratinocytes in the skin)	CCL27	CCR10	Serum	Human	Decreased in the serum of BMS-COPD subjects compared to controls	[77]
T lymphocytes, monocytes	Mature dendritic cells, endothelial cells	CX3CL1 (Fractalkin)	CX3CR1	Serum	Human	Associated with emphysema in chinese COPD patients	[89]

COPD: chronic obstructive pulmonary disease; CS: cigarette smoke; IP: interstitial pneumonia; BM-MSCs: bone marrow mesenchymal stem cells; BALF: broncho-alveolar lavage fluid; BMS: biomass smoke; EPCs: endothelial progenitor cells, HPCs: hematopoietic stem cells, SNP: single nucleotide polymorphism. Expressing cells are from the systemic circulation or lung cells (other organs are not mentioned) and based on human available data. Additional references for chemokines receptors [24] and attracted cells [90].

## Abbreviation

AIDS	Acquired immune deficiency syndrome
AC	Adenylate cyclase
BALF	Broncho-alveolar lung fluid
BM-MSCs	Bone marrow-mesenchymal stem cells
CC16	Club cell-16
CDK9	Cyclin-dependent kinase 9
COPD	Chronic obstructive pulmonary disease
CS	Cigarette smoke
CSE	Cigarette smoke extract
CT	Computed tomography
DAMPs	Damage-associated molecular patterns
DPPIV	Dipeptidyl peptidase-4
ECLIPSE	Evaluation of COPD Longitudinally to Identify Predictive Surrogate End-points
ECM	Extracellular matrix
EPCs	Endothelial progenitor cells
FDA	Food and drug administration
FEV1	Forced expiratory volume in one second
FEV1/FVC FLAME	Forced expiratory volume in one second/forced volume vital capacity EFfect of Indacaterol Glycopyronium Vs Fluticasone Salmeterol on COPD Exacerbations
GAGs	Glycosaminoglycans
GDP	Guanosine-5′-diphosphate
GPCRs	G-protein coupled receptors
GTP	Guanosine-5′-triphosphate
16-HBE	16-human bronchial epithelial cells
HMGB1	High mobility group box 1 protein
HIV	Human immunodeficiency virus
HPCs	Hematopoietic progenitor cells
IFN-γ IMPACT	Interferon-γ InforMing the PAthway of COPD Treatment
IRF3	IFN regulatory factor III
IL	Interleukine
IP3	Inositol trisphosphate
JAK/STAT	Janus kinase/signaling transducer and activator of transcription
kDa	kilo Daltons
LPS	Lipopolysaccharide
MAPK/Erk	Mitogen activated protein kinase/extracellular signal-regulated kinase
MCP-1	Monocyte chemotactic protein 1
MMP	Matrix metalloproteinase
PBMC	Peripheral blood mononucleated cells
PI3K	Phosphoinositide 3-kinase
PKC	Protein kinase C
PLC	Phosphoinositide-specific phospholipase C
PPE	Porcine pancreatic elastase
PRRs	Pattern recognition receptors
SNP	Single nucleotide polymorphism
SP-D	Surfactant protein D
sRAGE SUMMIT	Soluble receptor for advanced glycation end-products Study to Understand Mortality and Morbidity in COPD
TNF-α TORCH	Tumor necrosis factor-α TOwards a Revolution in COPD Health
TSG-6 UPLIFT	TNF-stimulated gene/protein-6 Understanding Potential Long-term Impacts on Function with Tiotropium
VEGF	Vascular endothelial growth factor
